# Concurrent Antidepressant Use and Metastasis Risk Among Women Receiving Tamoxifen for Breast Cancer: A Real‐World Cohort Study

**DOI:** 10.1002/pon.70617

**Published:** 2026-09-30

**Authors:** Shaimaa Elshafie, Mohammed Zuber, Ainhoa Gomez‐Lumbreras, Daniel C. Malone, Lorenzo Villa Zapata

**Affiliations:** ^1^ Department of Clinical and Administrative Pharmacy College of Pharmacy University of Georgia Athens Georgia USA; ^2^ Department of Epidemiology Rollins School of Public Health Emory University Atlanta Georgia USA; ^3^ Department of Pharmacotherapy College of Pharmacy University of Utah Salt Lake City Utah USA

**Keywords:** antidepressant, breast cancer, cancer outcomes, CYP2D6, depression, drug–drug interaction, metastasis, oncology, tamoxifen

## Abstract

**Objective:**

Concurrent use of antidepressants, particularly cytochrome P450 2D6 (CYP2D6) inhibitors, may reduce tamoxifen bioactivation and effectiveness. However, the clinical significance of this potential interaction remains inconclusive. This study examined whether concurrent antidepressant use is associated with increased metastasis risk among women receiving tamoxifen for nonmetastatic breast cancer.

**Methods:**

We conducted a cohort study using the Merative MarketScan Database. Eligible women with nonmetastatic breast cancer were followed from tamoxifen initiation until metastasis, disenrollment, or study end, whichever occurred first. Patients were classified as concurrent users (tamoxifen with overlapping antidepressant prescriptions) or nonusers (tamoxifen only). Time‐dependent Cox models estimated metastasis risk. Analyses were stratified by concurrent exposure duration and CYP2D6 inhibition degree and adjusted for relevant covariates.

**Results:**

Among 14,771 women (mean age: 51 ± 10 years), 4780 (32.4%) used tamoxifen concurrently with an antidepressant, most commonly moderate inhibitors (*n* = 1815) and least commonly strong inhibitors (*n* = 427). Median overlap duration was 8 months (IQR: 3–20). During follow‐up, 443 patients (3.0%) developed metastasis: 161 of 4780 concurrent users (3.4%) versus 282 of 9991 nonusers (2.8%). Concurrent antidepressant use was not significantly associated with metastasis risk (HR = 0.99; 95% CI: 0.81–1.21). Exposure duration was also not significantly associated with metastasis. Use of mixed‐inhibitor regimens was associated with higher risk in partially adjusted models (HR = 1.39; 95% CI: 1.04–1.87), though full adjustment attenuated the association.

**Conclusions:**

Concurrent antidepressant use was not significantly associated with increased metastasis risk among women receiving tamoxifen for nonmetastatic breast cancer. Individualized and coordinated decisions regarding antidepressant selection may help balance cancer control goals with the psychological and symptomatic management needs in this population.

## 1 Background

Breast cancer remains the most commonly diagnosed cancer among women in the United States, with an estimated 310,720 new cases projected in 2024 alone [1]. For patients with hormone receptor‐positive early‐stage breast cancer, endocrine therapy, including tamoxifen and aromatase inhibitors, has long been a cornerstone of treatment to reduce recurrence and improve survival outcomes [2]. Tamoxifen, a selective estrogen receptor modulator (SERM), exerts its therapeutic effects through active metabolites, particularly endoxifen, which are primarily generated via hepatic metabolism by the cytochrome P450 2D6 (CYP2D6) enzyme [3, 4].

Concurrent use of antidepressants is common during breast cancer treatment for managing depression, anxiety, and tamoxifen‐related symptoms such as hot flashes [5, 6, 7]. Depression and anxiety affect approximately 32%–42% of breast cancer patients and may adversely affect quality of life, treatment adherence, and overall survivorship [5, 6, 7]. However, antidepressant selection for women receiving tamoxifen may present an additional clinical consideration. Certain antidepressants, including paroxetine, fluoxetine, and bupropion, strongly inhibit CYP2D6 and may reduce tamoxifen bioactivation, raising concerns about a potential reduction in its therapeutic benefit [3, 4, 8].

This potential drug‐drug interaction creates a clinical dilemma for patients and their care teams. Avoiding antidepressants with CYP2D6‐inhibiting activity may reduce concerns about compromising tamoxifen effectiveness, but unnecessarily limiting antidepressant options could interfere with appropriate treatment of depression, anxiety, or treatment‐related symptoms. These considerations are particularly relevant during long‐term breast cancer care, when psychological symptom management and recurrence prevention must be balanced [9]. Although the potential interaction has prompted numerous clinical, pharmacokinetic, and pharmacogenomic investigations [10, 11], its clinical significance remains inconclusive and uncertain. Some studies have reported increased recurrence or mortality associated with CYP2D6 inhibitor use or poor metabolizer phenotypes, whereas others have shown no association [10, 12, 13, 14, 15, 16, 17]. Many of these studies have been limited by small sample sizes, methodological heterogeneity, and inadequate adjustment for important confounders [9, 18].

To address these gaps, we conducted a retrospective cohort study using the Merative MarketScan Research Database to examine whether concurrent antidepressant use is associated with an increased risk of breast cancer metastasis among women receiving tamoxifen for nonmetastatic breast cancer. We placed particular emphasis on the duration of concurrent exposure and degree of CYP2D6 inhibition. By applying time‐varying exposure modeling and stratified analyses, we aimed to advance the understanding of the real‐world clinical implications of this potential drug‐drug interaction and provide evidence that may inform individualized, multidisciplinary decisions regarding antidepressant selection during tamoxifen therapy.

## 2 Methods

### 2.1 Data Source

We used data from the Merative MarketScan Research Databases, including the Commercial Claims and Encounters (2017–2023), Medicare Supplemental and Coordination of Benefits (2017–2023), and Multi‐State Medicaid (2017–2022) files. These de‐identified administrative claims databases contain longitudinal data on healthcare services, including inpatient, outpatient, and prescription drug claims for diverse populations across the United States. The study was deemed not human subjects research by the University of Georgia Institutional Review Board because it relied entirely on de‐identified patient data.

### 2.2 Study Design

We conducted a retrospective cohort study following the STROBE (Strengthening the Reporting of Observational Studies in Epidemiology) reporting guidelines [19]. The index date was defined as the initiation of tamoxifen therapy following the earliest recorded diagnosis of breast cancer. Follow‐up began on the index date and continued until the first occurrence of metastasis, insurance disenrollment, or the end of the study period, whichever occurred first. A 6‐month pre‐index period was used to assess baseline comorbidities and confirm continuous insurance enrollment.

### 2.3 Inclusion and Exclusion Criteria

We included women who met all the following criteria: (i) diagnosis of nonmetastatic breast cancer; (ii) initiation of tamoxifen therapy after cancer diagnosis; and (iii) continuous insurance enrollment for at least 6 months prior to diagnosis. Patients were excluded if they discontinued tamoxifen therapy (i.e., had no prescription refills) for more than 180 consecutive days prior to a documented diagnosis of metastasis to reduce confounding related to treatment discontinuation.

### 2.4 Study Variables

Exposure to tamoxifen and antidepressants was identified using National Drug Codes (NDCs) from pharmacy claims. Antidepressants were categorized by CYP2D6 inhibition degree as follows: (i) strong inhibitors: paroxetine, fluoxetine, bupropion; (ii) moderate inhibitors: duloxetine, sertraline, citalopram, escitalopram, doxepin; and (iii) weak inhibitors: venlafaxine, desvenlafaxine, fluvoxamine, vortioxetine, vilazodone, levomilnacipran [7]. Concurrent users were defined as patients with any overlapping prescriptions for tamoxifen and an antidepressant during the study period; nonusers received tamoxifen alone. The outcome of interest was metastasis diagnosis identified using ICD‐10‐CM codes C77.x, C78.x, C79.x, and C80.0.

### 2.5 Statistical Analyses

Descriptive statistics were used to summarize demographic and clinical characteristics. Differences in baseline characteristics between concurrent users and nonusers were assessed using chi‐square tests for categorical variables and independent *t*‐tests or Wilcoxon rank‐sum tests for continuous variables, as appropriate.

Cox proportional hazards regression was used to estimate unadjusted and adjusted hazard ratios (HRs) with 95% confidence intervals (CIs) for metastasis, with antidepressant exposure modeled as a time‐varying covariate to account for changes in exposure status over time and minimize immortal time bias. The final adjusted model included the following covariates: age at tamoxifen initiation (categorized into five groups), geographic region, health insurance type (commercial, Medicare, or Medicaid), calculated Charlson Comorbidity Index (CCI) [20, 21], and time from cancer diagnosis to tamoxifen initiation. The primary hypothesis tested whether the hazard of metastasis differed between concurrent users and nonusers over time.

Subgroup analyses were conducted by duration of overlap (dichotomized at the median) and by CYP2D6 inhibition degree (strong, moderate, weak or mixed). The latter indicates sequential or combined exposure to antidepressants of differing inhibition degrees. Metastasis‐free survival was visualized using cumulative incidence curves, with nonusers as the reference group. All statistical analyses were performed using SAS software, version 9.4 TS1M6 (SAS Institute Inc., Cary, NC). A two‐sided *p*‐value ≤ 0.05 was considered statistically significant.

## 3 Results

### 3.1 Cohort Selection and Characteristics

Among 371,279 women with a diagnosis of breast cancer in the Merative MarketScan Research Databases, 14,771 patients met the inclusion criteria and comprised the analytical cohort (Figure 1). The primary reasons for exclusion included absence of tamoxifen prescriptions (*n* = 204,382; 55.0%), insufficient enrollment history before diagnosis (*n* = 139,866; 37.7%), or initiation of tamoxifen therapy prior to breast cancer diagnosis (*n* = 5833; 1.6%). Additional exclusions were made for evidence of metastasis before or within 3 months of tamoxifen initiation (*n* = 5098) and discontinuation of tamoxifen therapy for more than 6 months (*n* = 1329).

**FIGURE 1:**
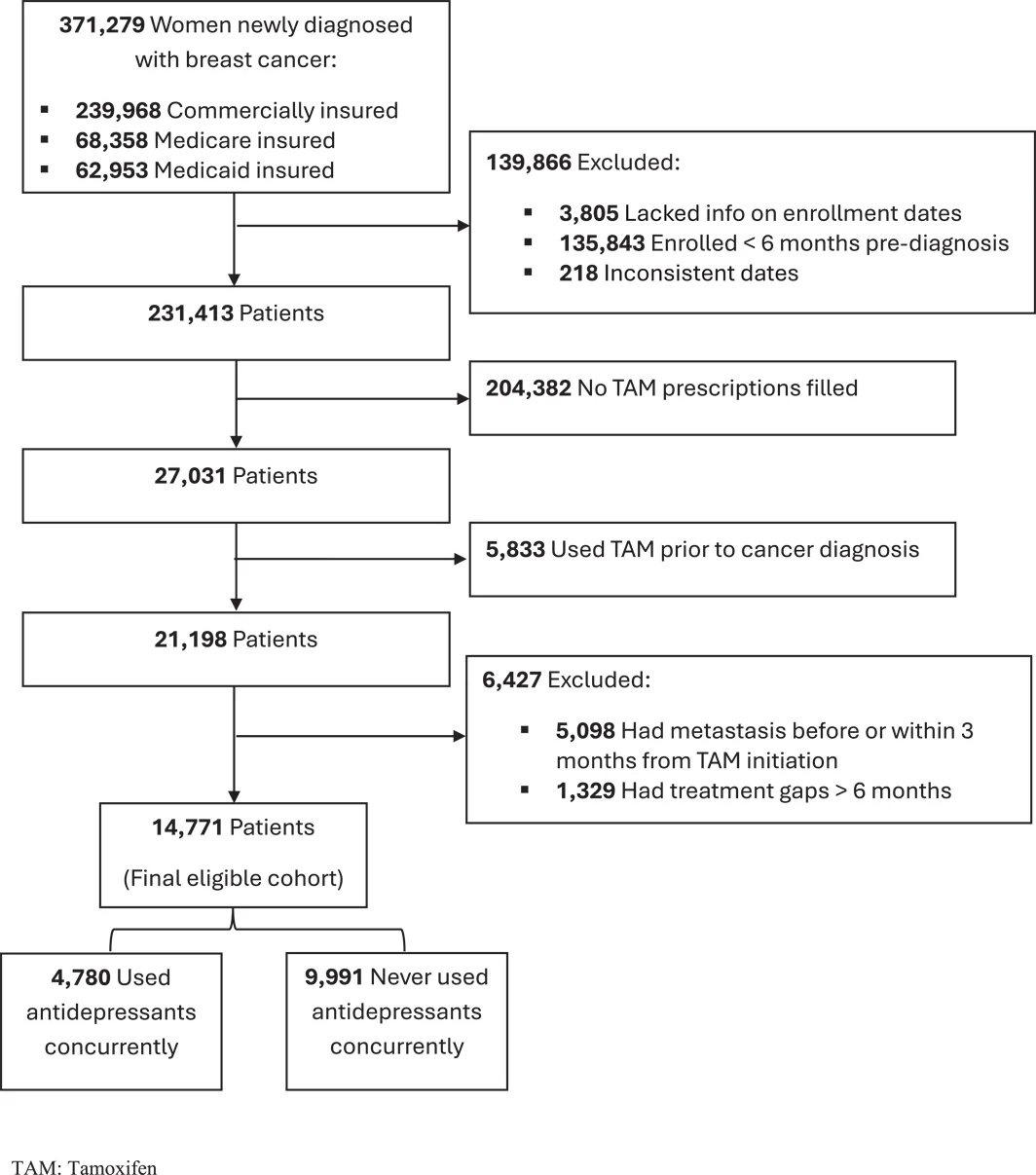
Flowchart of the study cohort creation.

The final cohort had a mean age of 51 years (SD = 10), with nearly half of the patients aged 45 to 54. Only 2.2% were younger than 35, and 7.2% were older than 64. The southern U.S. was the most represented region (*n* = 5962; 40.4%). Most patients were commercially insured (*n* = 12,570; 85.1%) and had moderate comorbidity burden, with a CCI score of 2 or 3 in over 90% of cases. Approximately 75% initiated tamoxifen therapy within 7 months of diagnosis, and the median treatment duration was 10 months (IQR: 4–22). Estrogen receptor‐positive tumors were documented in approximately 88% of the cohort.

A total of 4780 patients (32.4%) used tamoxifen concurrently with an antidepressant. The most commonly prescribed agents were venlafaxine (*n* = 1938/4780; 40.5%) and citalopram (*n* = 1396/4780; 29.2%). Among concurrent users, 72.7% (*n* = 3476) received a single antidepressant, and 22.7% (*n* = 1084) received two different agents. Based on CYP2D6 inhibition degree, most were prescribed moderate inhibitors (*n* = 1815; 38.0%), while strong inhibitors were least common (*n* = 427; 8.9%). The median duration of overlapping tamoxifen and antidepressant use was 8 months (IQR: 3–20).

Antidepressant use varied significantly across demographic and clinical subgroups. Compared with nonusers, concurrent users were more likely to be younger, reside in the South, have Medicaid insurance, higher CCI scores, longer tamoxifen treatment duration, and delayed initiation of tamoxifen post‐diagnosis. Baseline characteristics stratified by antidepressant use are presented in Table 1.

**TABLE 1:** Baseline cohort characteristics by concurrent use of antidepressants.

Characteristic^a^	Concurrent antidepressant user		Total (*N* = 14,771)
	Yes (*n* = 4780)	No (*n* = 9991)	
Age at TAM initiation (years)			
Mean ± SD	51 ± 9	52 ± 10	51 ± 10
Age group			
< 35	108 (2.3%)	223 (2.2%)	331 (2.2%)
35–44	1003 (21.0%)	1983 (19.8%)	2986 (20.2%)
45–54	2437 (51.0%)	4862 (48.7%)	7299 (49.4%)
55–64	979 (20.5%)	2113 (21.1%)	3092 (20.9%)
> 64	253 (5.3%)	810 (8.1%)	1063 (7.2%)
Region			
South	2088 (43.7%)	3874 (38.8%)	5962 (40.4%)
North Central	918 (19.2%)	1977 (19.8%)	2895 (19.6%)
Northeast	639 (13.4%)	1920 (19.2%)	2559 (17.3%)
West	591 (12.4%)	1507 (15.1%)	2098 (14.2%)
Unknown	544 (11.4%)	713 (7.1%)	1257 (8.5%)
Insurance type			
Commercial	4013 (84.0%)	8557 (85.6%)	12,570 (85.1%)
Medicaid	543 (11.4%)	694 (6.9%)	1237 (8.4%)
Medicare	224 (4.7%)	740 (7.4%)	964 (6.5%)
Charlson comorbidity index			
Mean score ± SD	3 ± 1	2 ± 1	2 ± 1
Time to initiate TAM (months)			
Median (IQR)	4 (2–7)	4 (2–6)	4 (2–7)
Time on TAM (months)			
Median (IQR)	12 (5–24)	9 (3–20)	10 (4–22)

*Note:* All values are expressed as number (%) unless otherwise noted.

Abbreviations: CCI: Charlson Comorbidity Index; IQR: interquartile range; SD: standard deviation; TAM: tamoxifen.

^a^ Differences in baseline characteristics between concurrent users and nonusers were statistically significant for all comparisons (*p* < 0.0001).

### 3.2 Metastasis Incidence

During follow‐up, 443 patients (3.0%) developed metastasis, including 161 of 4780 concurrent users (3.4%) versus 282 of 9991 nonusers (2.8%). The median time to metastasis was 17 months (IQR: 7–30). In time‐dependent Cox proportional hazards models, concurrent antidepressant use was not associated with increased metastasis risk in both the unadjusted model (HR = 1.06; 95% CI: 0.87–1.30; *p* = 0.5372) and adjusted models. Five‐year cumulative incidence curves (Figure 2) demonstrated similar metastasis‐free survival between concurrent users (6.7%) and nonusers (6.0%), with overlapping confidence intervals.

**FIGURE 2:**
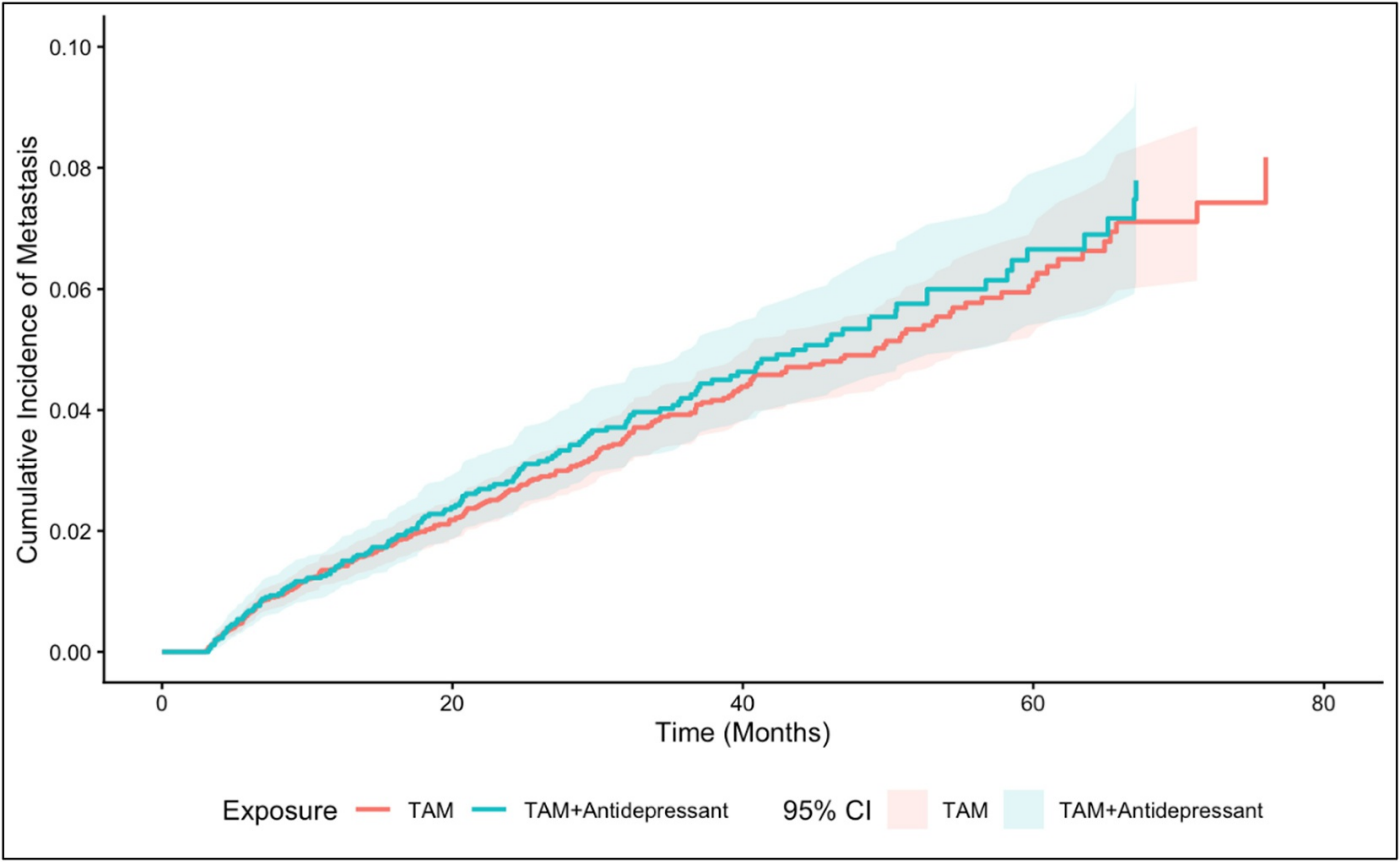
Cumulative metastasis incidence by antidepressant exposure.

Duration of concurrent antidepressant exposure (< 8 months vs. ≥ 8 months) was also not associated with differences in metastasis risk. At 5 years, the cumulative incidence remained comparable between long‐term users and nonusers. For short‐term users, 5‐year incidence was not reached during the study period (Supporting Information S1: Figure 1).

When stratified by CYP2D6 inhibition degree, overall differences in metastasis risk were not statistically significant (unadjusted model: *p* = 0.0581; adjusted model a: *p* = 0.0807; fully adjusted model: *p* = 0.1067). Weak and moderate inhibitors showed no risk association. However, concurrent use of mixed‐inhibitor regimens was associated with a significantly increased metastasis risk in the unadjusted model (HR = 1.39; 95% CI: 1.04–1.87; *p* = 0.0271) and partially adjusted model (HR = 1.36; 95% CI: 1.01–1.83; *p* = 0.0402), though the association attenuated and became non‐significant after full adjustment. At 5 years, users of mixed inhibitors had a slightly higher cumulative incidence of metastasis (8.6%) compared with nonusers (6.0%) (Supporting Information S1: Figure 2). Risk estimates for strong inhibitors were imprecise due to the small sample size of this subgroup. Full results from the Cox regression models are presented in Table 2.

**TABLE 2:** Results of unadjusted and adjusted Cox models.

Exposure group	Unadjusted model		Adjusted model^a^		Adjusted model^b^	
	HR (95% CI)	*p*‐value	HR (95% CI)	*p*‐value	HR (95% CI)	*p*‐value
Exposure						
TAM alone (ref.)	—	—	—	—	—	—
TAM + antidepressant	1.06 (0.87–1.30)	0.5372	1.06 (0.87–1.29)	0.5649	0.99 (0.81–1.21)	0.9469
Exposure duration		0.0895		0.1317		0.3479
None (ref.)	—	—	—	—	—	—
< 8 months	1.29 (0.99–1.66)	0.0568	1.26 (0.97–1.63)	0.0815	1.13 (0.87–1.47)	0.3506
≥ 8 months	0.92 (0.71–1.18)	0.4920	0.92 (0.72–1.19)	0.5285	0.89 (0.69–1.15)	0.3737
Inhibition degree		0.0581		0.0807		0.1067
None (ref.)	—	—	—	—	—	—
Strong	0.42 (0.18–1.02)	0.0559	0.42 (0.18–1.02)	0.0566	**0.38 (0.16–0.92)**	**0.0326**
Moderate	1.04 (0.77–1.40)	0.8154	1.02 (0.76–1.38)	0.8786	0.96 (0.71–1.30)	0.8028
Weak	0.98 (0.70–1.36)	0.8996	1.00 (0.72–1.40)	0.9881	0.96 (0.69–1.33)	0.7911
Mixed^c^	**1.39 (1.04–1.87)**	**0.0271**	**1.36 (1.01–1.83)**	**0.0402**	1.26 (0.94–1.70)	0.1240

*Note:* Bold font indicates statistical significance at the 0.05 threshold.

Abbreviations: CI: confidence interval; HR: hazard ratio; TAM: tamoxifen.

^a^ Model a was adjusted for age and Charlson Comorbidity Index (CCI) score.

^b^ Model b was additionally adjusted for geographic region, insurance type and time to tamoxifen initiation.

^c^ Mixed indicates sequential or combined exposure to antidepressants of differing inhibition degrees.

## 4 Discussion

In this large retrospective cohort study of women receiving tamoxifen for nonmetastatic breast cancer, we found no significant association between concurrent antidepressant use and the risk of developing metastasis. Across both unadjusted and adjusted time‐varying Cox models, patients receiving tamoxifen concurrently with antidepressants exhibited a similar risk of metastasis compared to those receiving tamoxifen alone. Additionally, the duration of concurrent antidepressant exposure, whether short‐ or long‐term, was not associated with differences in metastatic outcomes. When antidepressants were stratified by CYP2D6 inhibition degree, overall comparisons across inhibition categories were not statistically significant. However, the use of antidepressant regimens with mixed inhibition profiles was associated with a significantly increased metastasis risk in unadjusted and partially adjusted models; this association was attenuated and no longer significant in the fully adjusted analysis. The small number of patients exposed exclusively to strong inhibitors also resulted in imprecise risk estimates for this subgroup.

These findings may provide reassurance to patients and the broader psycho‐oncology professionals that clinically indicated treatment for depression, anxiety, and treatment‐related symptoms should not be withheld solely because of concerns about a potential interaction with tamoxifen. Our study adds to the growing, yet inconclusive, body of evidence on the clinical relevance of antidepressant use during tamoxifen therapy. Consistent with several prior observational studies, our results suggest no significant association between concurrent antidepressant use and breast cancer outcomes, including disease progression. For example, Haque and colleagues found no association between antidepressant use and the risk of any recurrence in a large cohort of 16,887 breast cancer patients within the California Kaiser Permanente system [15]. Similarly, an analysis of data from 4493 premenopausal Danish patients reported no significant association between the use of CYP450 inhibitors and local, contralateral or distant recurrence [16]. Another observational study of 960 patients also documented no increased risk of adverse clinical outcomes with concurrent CYP2D6 inhibitor use [17]. However, differences in the classification of CYP2D6 inhibitors across some prior studies may have contributed to heterogeneity in the available evidence and should be considered when interpreting these findings.

The absence of an observed association does not necessarily exclude a biologically relevant interaction. Pharmacokinetic and pharmacogenomic research has consistently linked reduced CYP2D6 activity, whether due to strong CYP2D6 inhibitors such as paroxetine or poor‐metabolizer genotypes, with lower endoxifen levels and compromised treatment efficacy [22, 23, 24, 25]. A recent meta‐analysis of 33 studies further revealed that CYP2D6 polymorphisms, particularly poor‐metabolizer status, were associated with the highest rates of cancer recurrence and mortality [11]. Thus, the biological plausibility of a tamoxifen–antidepressant interaction remains important even when its clinical effect is difficult to detect in observational studies.

Several factors may contribute to the difficulty in translating this pharmacologic relationship into observable clinical outcomes. Tamoxifen bioactivation involves multiple CYP450 enzymes in addition to CYP2D6, as well as phase II conjugation pathways and transporters (e.g., ABC transporters) [26]. The contribution of these metabolic pathways may vary according to patient characteristics and concomitant medications [26]. Consequently, altered activity in a single pathway (such as CYP2D6 inhibition) may not translate directly into a clinically significant reduction in tamoxifen effectiveness or a measurable difference in long‐term cancer outcomes. Future approaches that integrate pharmacogenetic information, clinical characteristics, medication exposure, and other determinants of tamoxifen metabolism may help address this complexity by supporting personalized management strategies and identifying patients who are more susceptible to clinically meaningful drug–drug interactions.

Medication adherence represents another important consideration when assessing tamoxifen clinical effectiveness. Individuals with higher CYP2D6 activity, and therefore greater production of endoxifen, may experience more side effects, potentially increasing the risk of nonadherence [27]. In addition, administration of over‐the‐counter supplements for tamoxifen‐induced side effects (e.g., St. John’s wort commonly used for menopausal symptoms) can markedly alter tamoxifen‐metabolizing enzyme activity but are not captured in claims data [18]. These factors make it difficult to distinguish pharmacologic effects from differences in patient behavior and medication use in real‐world studies.

The long latency between changes in tamoxifen exposure or metabolism and clinically apparent cancer outcomes presents an additional challenge. Treatment failure may manifest months or years after an alteration in tamoxifen exposure, complicating causal inference and increasing the potential for residual confounding [28]. Furthermore, ethical and logistical constraints preclude the feasibility of prospective randomized trials assigning patients to tamoxifen with or without CYP2D6‐inhibiting antidepressants; thus, observational epidemiologic studies will remain crucial, although they are inherently limited by residual confounding from unmeasured factors [29]. In our cohort, for example, patients with more severe or treatment‐resistant depression may have been more likely to receive multiple or higher‐potency antidepressants, introducing confounding by indication that cannot be fully addressed in claims‐based analyses. The increased metastasis risk observed among patients exposed to mixed inhibitor regimens may therefore reflect differences in underlying patient characteristics, prescribing patterns, or depression severity rather than a direct pharmacologic effect. This interpretation is supported by attenuation of the association following full adjustment.

This study has several strengths. It leveraged a large real‐world cohort of tamoxifen‐treated patients, allowing evaluation of a clinically common medication combination in routine practice and enhancing the generalizability of its findings. Excluding patients with prolonged tamoxifen gaps reduced potential bias from treatment discontinuation and nonadherence, leading to more accurate and reliable risk estimates. Additionally, we applied time‐varying exposure modeling to account for the temporal dynamics of antidepressant exposure and thereby minimize immortal time bias and strengthen the validity of our Cox regression approach.

### 4.1 Study Limitations

Several limitations should be acknowledged. First, the absence of data on CYP2D6 genotype and detailed tumor characteristics limited our ability to account for important determinants of tamoxifen metabolism and disease prognosis. Second, prescription refill records indicate medication dispensing rather than actual consumption and therefore may not precisely reflect adherence. Third, claims data do not capture all factors that may influence antidepressant selection or tamoxifen effectiveness (e.g., depression severity, over‐the‐counter medications and supplements, and other behavioral or clinical factors), contributing to residual or unmeasured confounding. Fourth, the use of metastasis as the outcome of tamoxifen‐antidepressant interaction may not fully capture all clinically relevant forms of treatment failure, such as local and regional recurrences. Finally, metastasis detection depends on the intensity of follow‐up and surveillance practices, which may vary across patients and clinical settings.

### 4.2 Clinical Implications

These findings have practical implications for the multidisciplinary care of women receiving tamoxifen for nonmetastatic breast cancer. Decisions regarding antidepressant selection or modification during breast cancer treatment extend beyond cancer control and must also account for patients’ psychological and symptomatic needs. Potential tamoxifen–antidepressant interactions should be considered within this broader clinical context rather than automatically precluding appropriate antidepressant treatment or undertreating psychological and treatment‐related symptoms.

The biological plausibility of CYP2D6‐mediated interactions and ongoing clinical uncertainty surrounding specific antidepressant combinations support cautious and individualized prescribing. When clinically appropriate, antidepressants with strong CYP2D6 inhibitory activity may be avoided when similarly effective alternatives with less enzyme inhibition are available [30]. However, medication selection should also account for treatment indication, expected benefits, tolerability, prior treatment response, and patient preferences. Communication among patients, oncologists, psychosocial professionals, and other members of the care team is therefore important to ensure that concerns about potential drug–drug interactions do not create unnecessary barriers to psychiatric care. Coordinated, multidisciplinary decision‐making can help balance cancer control goals with effective management of psychological and treatment‐related symptoms.

## 5 Conclusions

In this large real‐world cohort, concurrent antidepressant use was not significantly associated with an increased risk of metastasis among women receiving tamoxifen for nonmetastatic breast cancer. However, these findings do not eliminate uncertainty given the biological plausibility of CYP2D6‐mediated interactions. Individualized and coordinated decisions across care teams regarding antidepressant selection during tamoxifen therapy may help balance cancer control goals with the psychological and symptomatic management needs of patients. Future studies incorporating pharmacogenetic information, clinical measures of mental health, and detailed longitudinal medication exposure are needed to determine whether specific patients are particularly vulnerable to clinically meaningful interactions.

## Author Contributions

**Shaimaa Elshafie:** conceptualization, methodology, formal analysis, interpretation of data, writing – original draft. **Mohammed Zuber:** conceptualization, methodology, interpretation of data, writing – review and editing. **Ainhoa Gomez‐Lumbreras:** writing – review and editing. **Daniel C. Malone:** writing – review and editing. **Lorenzo Villa Zapata:** conceptualization, methodology, interpretation of data, writing – review and editing, supervision.

## Funding

The authors have nothing to report.

## Ethics Statement

The authors have nothing to report.

## Conflicts of Interest

The authors declare no conflicts of interest.

## Supporting information


Supporting Information S1

## Data Availability

The data that support the findings of this study are available from Merative MarketScan. Restrictions apply to the availability of these data, which were used under license for this study. Data are available from the author(s) with the permission of Merative MarketScan.
