# Supplementary material for: Concurrent Antidepressant Use and Metastasis Risk Among Women Receiving Tamoxifen for Breast Cancer: A Real‐World Cohort Study
Source: Psychooncology. 2026 Sep 30;35(10):e70617. doi: 10.1002/pon.70617 (PMC13626258; doi:10.1002/pon.70617)
Supplement: Supplementary file 1 [file PON-35-e70617-s001.docx]

**Supplementary Figure 1: Cumulative Metastasis Incidence by Antidepressant Exposure Duration**

**Supplementary Figure 2: Cumulative Metastasis Incidence by Inhibitor Degree**
